# The impact of demographics and positioning on the imaging features of the optic nerve sheath and ophthalmic vessels

**DOI:** 10.1186/s13089-025-00403-x

**Published:** 2025-01-16

**Authors:** David Berhanu, Luís Abegão Pinto, Inês Carneiro, Isabel Fragata, Joana Tavares Ferreira, Lia Lucas Neto

**Affiliations:** 1 Faculdade de Medicina da Universidade de Lisboa, Lisbon, Portugal; 2 Serviço de Imagiologia Neurológica, Centro Hospitalar Universitário Lisboa Norte, Lisbon, Portugal; 3 Serviço de Oftalmologia, Centro Hospitalar Universitário Lisboa Norte, Lisbon, Portugal; 4 Serviço de Neurorradiologia, Centro Hospitalar Universitário Lisboa Central, Lisbon, Portugal; 5 NOVA Medical School – Faculdade de Ciências Médicas, Lisbon, Portugal

**Keywords:** Optic nerve sheath, Ultrasonography, Doppler, Ophthalmic artery, Central retinal artery, Optic nerve sheath diameter

## Abstract

**Background:**

There are significant discrepancies in the optic nerve sheath diameter (ONSD) reported in the literature. We aimed to determine the ultrasonographic imaging features of ONSD and ophthalmic vessels in a healthy population, using a standardized protocol, and to estimate the effect of demographics and positioning changes on imaging measurements.

**Methods:**

We measured the mean values of the ONSD in supine and sitting position and the Doppler imaging parameters of the ophthalmic, central retinal and short posterior ciliary arteries. Inter-observer reliability was assessed using intraclass correlation coefficient (ICC). Linear regression models were fitted to predict the effect of demographic and clinical determinants on the imaging features.

**Results:**

A total of 50 measurements were obtained for each observer. The mean ONSD was 5.9 mm and there was a mean reduction of 0.2 mm when assessed in sitting position (*p* < 0.001). Doppler analysis showed higher peak-systolic velocity and resistive index in the ophthalmic artery (35.6 cm/s vs. 12.0 cm/s; 0.78 vs. 0.70) compared to the central retinal artery (*p* < 0.001). Age, sex, heart rate and systolic blood pressure were significant determinants of the imaging features, with ONSD being larger in males (*p* < 0.001) and increasing with heart rate (*p* = 0.001). ICC estimates indicated ‘good’ inter-observer reliability of the ONSD and the ophthalmic and central retinal arteries velocities and resistance.

**Conclusions:**

Our findings suggest a significant impact of patient demographics and positioning during ultrasonography on the normal imaging features of the ONSD and ophthalmic vessels. The heterogeneity in methodology and clinical cohorts may justify previous discrepancies in the literature. These findings can assist in the interpretation of imaging features in clinical settings and in the standardization of point of care ONSD ultrasonography.

## Background

Ultrasonography of the retrobulbar compartment of the orbit can assess the optic nerve sheath diameter (ONSD) and the Doppler of the ophthalmic vessels (ophthalmic, central retinal and posterior ciliary arteries) [1–4]. The ONSD has been used as a marker for intracranial pressure (ICP) and it has an overall high sensitivity and specificity to detect increased ICP, according to previous meta-analysis [5–8]. However, significant differences exist in the definition of normal and increased ONSD, with cut-offs for increased ICP in the literature ranging from 4.1 to 7.2 mm [9, 10] and a systematic review concluding that, despite high sensitivity and specificity, a unified ONSD value is missing [11]. The lack of a consistent ONSD cut-off to diagnose increased ICP may limit its clinical application and a clearer definition of normal ONSD imaging features is necessary [12].

Previous studies on ONSD ultrasonography have used widely different protocols, with varying measurement techniques, patient positioning, probes, and sonographer’s specializations and duration of training. These differences are not neglectable, as the references used for measurement can impact the assessment of ONSD [13, 14]. Similarly, it is unclear how the degree of head elevation may affect ONSD, with one report suggesting that ONSD may be affected by positioning [15]. Overall, a standardized protocol of ultrasonographic assessment of ONSD and ophthalmic vessels, with strict measurement techniques and patient positioning is central to obtain valid and generalisable results [13]. This remains of critical importance as newly described indexes to predict ICP are still at least partially dependent on optic nerve sheath measurement [16, 17], highlighting the need to understand factors that impact the ONSD.

In addition to ONSD, Doppler ultrasound waveforms of the ophthalmic vessels have been proposed as sensors to detect increased ICP [18–21]. Analysis of its diagnostic accuracy revealed that the ophthalmic and central retinal arteries indices are only moderately accurate to detect increased ICP [19, 20]. However, as with the ONSD, extensive literature differences exist regarding normal velocities and resistive indices of the ophthalmic vessels [22–24]. Overall, little is known about the association of Doppler imaging of the ophthalmic vessels and the ONSD in the same population and which cut-off values to use as reference for healthy individuals and for the diagnosis of increased ICP [19, 20].

We aimed to determine the normal imaging features of optic nerve ultrasonography in healthy individuals, namely of ONSD and ophthalmic vessels, and to assess the effect of demographic and positioning changes on imaging measurements.

## Methods

### Study design and participants

We conducted a prospective cohort study of 25 healthy volunteers recruited at the Lisbon Academic Medical Centre. All included participants gave consent according to local ethic regulations. Exclusion criteria comprised: (1) age under 18; (2) previous neurological disorder; or (3) previous ophthalmological disorder, traumatic injury or surgery. The main outcome was to characterise the imaging features—ultrasonographic ONSD and Doppler waveform of ophthalmic vessels—in a healthy cohort using a standardized protocol. As secondary endpoints, we evaluated: (1) the inter-observer reliability of ultrasonographic measurements; (2) the effect of demographic and clinical determinants on the ONSD and Doppler parameters; (3) the effect of positioning in the ONSD measurement.

### Clinical data

We retrieved demographic and clinical data, including: age, sex, height, weight, body mass index, blood pressure and heart rate measured immediately before ultrasonographic examination, vascular risk factors (hypertension, diabetes, and smoking status), previous medical history. A standardized optic nerve ultrasonography was used to assess the ONSD and Doppler waveform indices of the ophthalmic, central retinal and ciliary arteries.

### Ultrasound protocol

Ultrasonographic examinations of the optic nerve were performed in B-mode to determine the ONSD using a linear array transducer probe (6.2–12.0 MHz) on a Toshiba Aplio 400 ultrasound system. Colour Doppler analysis of the ophthalmic vessels was executed to identify the ophthalmic vessels. The examinations were independently performed by two researchers (DB, LAP) with experience in optic nerve ultrasonography and blinded to each other’s examination.

Each participant was assessed in supine position with head at 0° and a waiting period of 3 min in that position was given before assessment. The participants were asked to look straight ahead with eyes closed and the ultrasonography was executed with the probe in the axial/transverse plane. Measurements of the ONSD and Doppler waveforms of the ophthalmic, central retinal, and short posterior ciliary arteries were taken from each eye.

Before their ultrasonographic examination in supine position, a subgroup of participants was also assessed in the sitting position immediately before laying down in supine position.

### Measurement technique

**Fig. 1:**
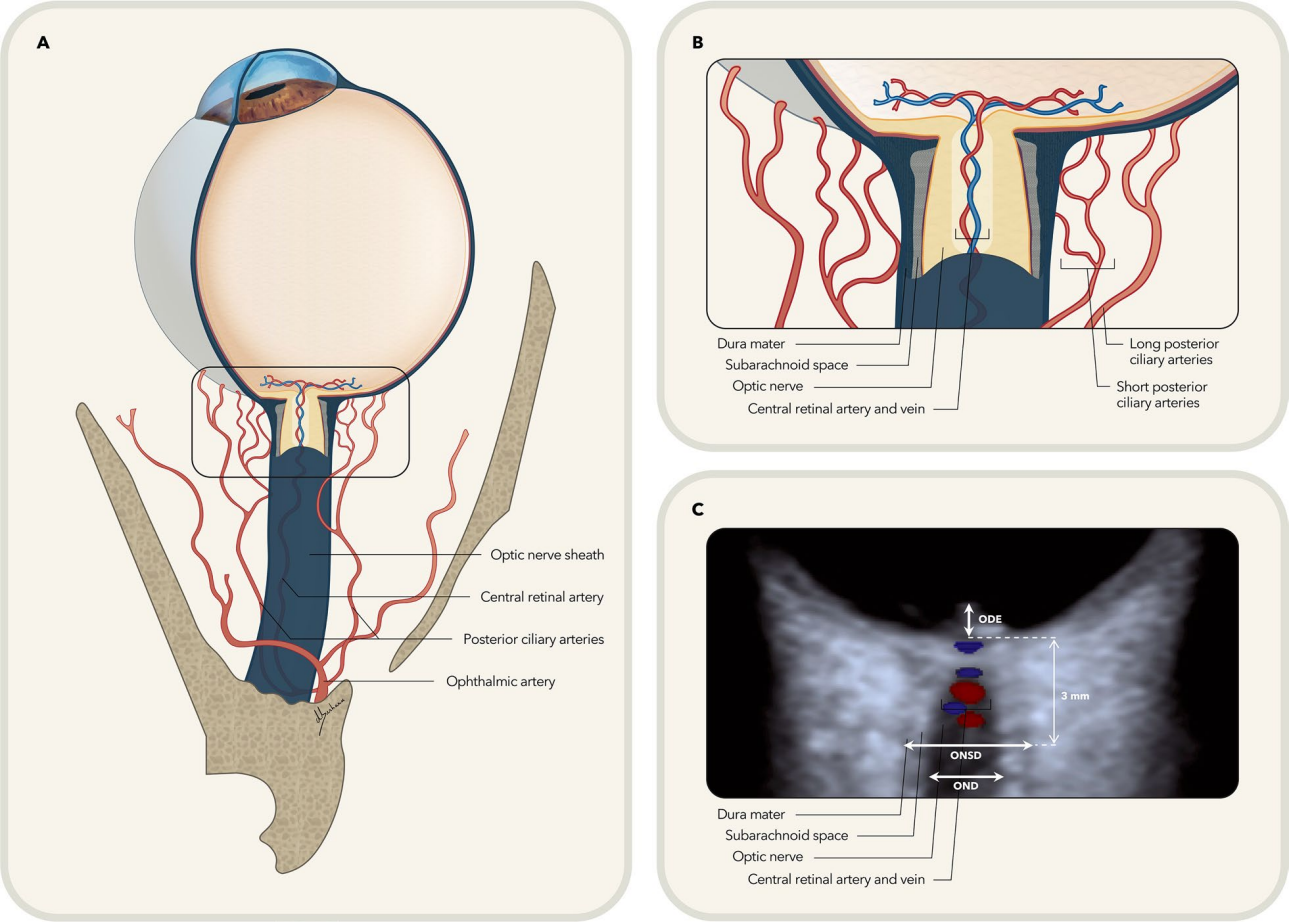
Anatomy and imaging of the optic nerve sheath and ophthalmic vessels. **A** Anatomy of the orbit portraying a schematic depiction of the eye, optic nerve and ophthalmic vessels. **B** Amplification of previous image showing an axial slice of the optic nerve sheath and the anatomic representation of the optic nerve and central retinal vessels surrounded by the subarachnoid space and meningeal layers. The short and long posterior ciliary arteries are also represented piercing the sclera near the optic nerve. **C** Optic nerve ultrasonography showing the corresponding imaging features to the anatomic structures observed in **B**. The optic nerve is identifiable as a dark central structure, where the coloured central retinal vessels are traversing (central retinal artery in red, central retinal vein in blue) The dura mater corresponds to the outer hypoechoic lines and the subarachnoid space is visualised as two stripped hyperechoic bands. The ONSD was measured 3 mm behind the lamina cribrosa/outer retinal rim in the axial plane as illustrated in the image. ODE, optic disk elevation; OND, optic nerve diameter; ONSD, optic nerve sheath diameter. *Source*: Adapted from Berhanu et al. [17]

An axial lengthwise optic nerve approach was used to visualize the lamina cribrosa, the optic disk and the optic nerve-sheath at its centre. Special attention was given to curvatures of the optic nerve, and, at this point, participants were asked to perform a slight horizontal gaze movement in order to align the optic nerve parallel with the transverse imaging plane. The ONSD was measured 3 mm behind the lamina cribrosa in the axial slice crossing the midpoint of the optic nerve to ensure accurate estimation of diameter. The limits of the optic nerve-sheath were carefully identified according to anatomical landmarks (Fig. 1). The optic nerve is identified as a central hypoechoic structure, surrounded by two stripped hyperechoic bands laterally, which correspond to the subarachnoid space. The outer hypoechoic lines visualized during the ultrasonography correspond to the dura-mater, and this meningeal layer matches up to the outer border of the ONSD. The ONSD was measured at the external margin of the dura-mater (Fig. 1C).

Doppler ultrasonography was performed using the same probe positioning and colour mode was used to identify the ophthalmic artery medially to the optic nerve-sheath, immediately after crossing the optic nerve (approximate depth of 4 mm) in an angle ranging between 0° and 30° (Fig. 1A). The central retinal artery was identified within the optic nerve together with the central retinal vein, in the same slice where the ONSD was measured. The short posterior ciliary arteries were identified laterally or medially to the optic nerve-sheath at the same depth as the measurement of the central retinal artery. The Doppler waveforms for the three arteries were obtained automatically at these specified points and the peak-systolic velocity (PSV), end-diastolic velocity (EDV), resistive index (RI) and early-systolic acceleration (ESA) were determined.

### Statistical analysis

Variables were described using mean and standard deviation or median and interquartile range (IQR), according to their distribution. Inter-rater reliability was assessed with intraclass correlation coefficient (ICC) based on a mean-rating, absolute-agreement, two-way random-effects model [25, 26]. ICC estimates and their 95% confidence intervals (CI) were determined. We performed a multivariate linear regression model to determine the effect of demographic data (age, sex, height, weight, body mass index) and clinical data (systolic and diastolic blood pressure, heart rate) on the ONSD and Doppler imaging parameters with a good inter-rater reliability, followed by a stepwise process to optimize the regression model. Variables with small numbers (*n* ≤ 6) were not included in the model to avoid overfitting. After testing for normality, analysis between subgroups were performed using a 2-tailed paired *t* test. Statistical significance was set at *p* values <0.05. All analysis were performed using Stata version 16.1 (StataCorp. 2019. Stata Statistical Software: Release 16. College Station, TX: StataCorp LLC).

## Results

Of the 25 participants included in the cohort, 14 participants were female. The mean age was 27 (IQR 22–41) and the mean body mass index was 23 (IQR 21–25). Two participants had a previous history of hypertension and were taking anti-hypertensive medication. At the time of the ultrasonographic examination, both participants had normal blood pressure measurements. Demographic and clinical characteristics of the cohort are described in Table 1.

**Table 1:** Demographic and clinical characteristics of the cohort

Age (years)	27 [22–41]
Female sex	14 (56%)
Height (cm)	169 [160–178]
Weight (kg)	68 [59–75]
Body mass index	23 [21–25]
Systolic blood pressure (mmHg)	120 [115–125]
Diastolic blood pressure (mmHg)	74 [69–81]
Heart rate (bpm)	67 [58–80]
Hypertension	2 (8%)
Diabetes	0 (0%)
Smoking	4 (16%)

Data are presented as median [IQR] or *n* (%)

*cm* centimetres, *kg* kilograms, *mmHg* millimetres of mercury, *bpm* beats per minute

### Normal imaging features of ONSD and Doppler waveform of ophthalmic vessels (ophthalmic, central retinal and short posterior ciliary arteries)

**Fig. 2:**
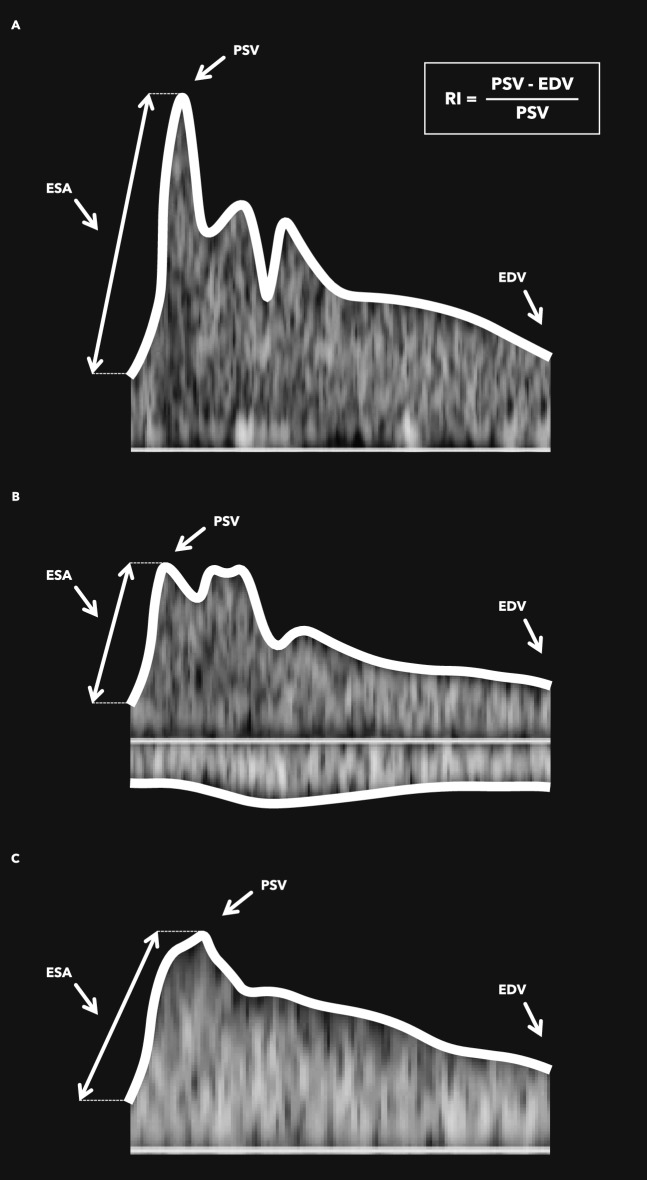
Doppler waveforms of the ophthalmic vessels. **A** Doppler of the ophthalmic artery showing a higher PSV and ESA compared with the central retinal artery (**B**) and short posterior ciliary artery (**C**). There is also a greater difference between PSV and EDV resulting in a higher RI in the ophthalmic artery compared to the other vessels in the orbit. **B** Doppler of the central retinal artery, which also portrays the central retinal vein, as these vessels have an analogous trajectory inside the optic nerve sheath. The central retinal vein waveform is located below the reference line, due to the inverse flow direction of venous drainage. **C** Doppler of the short posterior ciliary artery showing slower velocities, resistance, and acceleration. EDV, end-diastolic velocity; ESA, early-systolic velocity; PSV, peak-systolic velocity; RI, resistive index

A total of 50 measurements were performed by each researcher. The mean ONSD was 5.9 mm (95% CI 5.8–6.0 mm). Additionally, Doppler imaging of the ophthalmic artery revealed a waveform with high velocity and acceleration (PSV: 35.6 cm/s; EDV: 7.4 cm/s; ESA: 483.3 cm/s^2^), and a high mean resistance (RI: 0.78). The central retinal and short posterior ciliary arteries had slower mean velocities and acceleration (PSV: 11.9 cm/s and 10.4 cm/s; EDV: 3.5 cm/s and 3.6 cm/s; ESA: 135.1 cm/s^2^ and 91.6 cm/s^2^) and lower RI (0.70 and 0.64), compared to the ophthalmic artery (*p* < 0.001, Fig. 2).

The complete imaging features of ultrasonographic ONSD and Doppler waveform analysis, including confidence intervals for mean values, are reported in Table 2.

**Table 2:** Normal imaging features of ONSD and Doppler waveform of ophthalmic vessels

	Imaging features		
	Mean	SE	95% CI
ONSD (mm)	5.9	0.1	5.8–6.0
Ophthalmic artery			
PSV (cm/s)	35.6	1.2	33.1–38.0
EDV (cm/s)	7.4	0.4	6.7–8.2
RI	0.78	0.01	0.77–0.80
ESA (cm/s^2^)	483.3	26.8	429.3–537.3
Central retinal artery			
PSV (cm/s)	11.9	0.5	11.0–12.9
EDV (cm/s)	3.5	0.2	3.2–3.9
RI	0.70	0.01	0.68–0.72
ESA (cm/s^2^)	135.1	8.6	117.9–152.2
Short posterior ciliary artery			
PSV (cm/s)	10.4	0.4	9.5–11.2
EDV (cm/s)	3.6	0.2	3.2–3.9
RI	0.64	0.01	0.62–0.66
ESA (cm/s^2^)	91.6	6.3	78.9–104.2

The imaging features column represents the mean value obtained from all measurements and the 95% confidence intervals for the respective mean value, using standard errors

*ONSD* optic nerve-sheath diameter, *PSV* peak systolic velocity, *RI* resistive index, *ESA* early systolic acceleration, *mm* millimetres, *cm* centimetres, *s* second

### Reliability of optic nerve ultrasonographic examination

There was a ‘good to excellent’ inter-observer reliability for ONSD assessment (ICC 0.87, 95% CI 0.76–0.92). Reliability of Doppler imaging varied according to vessel and waveform parameter being assessed (Table 3). We found ICC estimates indicating ‘good’ reliability of the PSV of all the ophthalmic vessels (0.79–0.88), with the 95% CI showing a ‘moderate to excellent’ reliability of PSV. Similarly, the ICC estimates of the EDV and RI of the ophthalmic and central retinal arteries also showed a ‘good’ reliability between raters (RI: 0.82 and 0.84, respectively), with a wide 95% CI ranging from ‘moderate to excellent’. Overall, the ESA had ‘moderate’ ICC estimates for inter-observer reliability. From all the ophthalmic vessels, the short posterior ciliary artery had the lower inter-observer reliability for all the Doppler waveform indices (Table 3).

**Table 3:** Inter-observer reliability analysis of the ultrasonographic measurements using ICC

	Imaging features		Inter-observer agreement	
	Rater 1	Rater 2	ICC	95% CI
ONSD (mm)	5.93 (0.58)	5.85 (0.54)	**0.87**	**0.76–0.92**
Ophthalmic artery				
PSV (cm/s)	35.3 (8.7)	35.9 (10.3)	**0.86**	**0.67–0.94**
EDV (cm/s)	7.6 (3.0)	7.2 (2.8)	**0.76**	**0.51–0.88**
RI	0.78 (0.06)	0.78 (0.08)	**0.82**	**0.64–0.91**
ESA (cm/s^2^)	497.9 (171.7)	468.7 (201.9)	0.69	0.20–0.88
Central retinal artery				
PSV (cm/s)	12.4 (3.6)	11.5 (3.9)	**0.88**	**0.74–0.94**
EDV (cm/s)	3.7 (1.3)	3.4 (1.5)	**0.77**	**0.51–0.90**
RI	0.69 (0.08)	0.71 (0.07)	**0.84**	**0.63–0.93**
ESA (cm/s^2^)	135.7 (54.2)	134.5 (71.1)	0.65	0.20–0.84
Short posterior ciliary artery				
PSV (cm/s)	10.0 (2.9)	10.4 (3.0)	**0.79**	**0.51–0.91**
EDV (cm/s)	3.5 (1.4)	3.7 (1.3)	0.59	0.05–0.82
RI	0.65 (0.07)	0.64 (0.06)	0.65	0.04–0.87
ESA (cm/s^2^)	83.7 (32.6)	98.8 (54.5)	0.58	0.06–0.81

Data are presented as mean (SD). ICC with 95% CI are presented for an absolute agreement, mean-measurement, two-way random effects model

Highlighted values represent ICC central estimates indicative of ‘good’ or ‘excellent’ reliability

*ICC* intraclass correlation coefficient, *ONSD* optic nerve-sheath diameter, *PSV* peak systolic velocity, *RI* resistive index, *ESA* early systolic acceleration

### Effect of demographic and clinical determinants on the ONSD and Doppler waveform of ophthalmic vessels

In a univariate regression analysis, age, sex and heart rate were significantly associated with the ONSD, with females having smaller diameters (*β* = −0.40 mm, *p* = 0.015) and larger ONSD being associated with increasing age (*β* = 0.13 mm/10-years, *p* = 0.043) and heart rate (*β* = 0.12 mm/10 bpm, *p* = 0.042). In the multivariate model, the effect of sex and heart rate was still significantly associated with the ONSD, but age lost its significant effect (Table 4).

**Table 4:** Summary of the effect demographic and clinical data on the ONSD and Doppler waveform of ophthalmic vessels

Model parameters	ONSD (mm)			Ophthalmic artery (RI)			Central retinal artery (RI)		
	*β*	95% CI	*p*	*β*	95% CI	*p*	*β*	95% CI	*p*
Age, ±10-years	−0.07	−0.21 to 0.07	0.327	−**0.03**	−**0.06 to **−**0.01**	**0.007**	0.01	−0.03 to 0.04	0.758
Female sex	−**1.36**	−**1.99 to **−**0.74**	**<0.001**	0.12	−0.07 to 0.09	0.767	−**0.15**	−**0.27 to **−**0.03**	**0.015**
Weight (kg)	−0.11	−0.22 to 0.01	0.068	−0.01	−0.01 to 0.01	0.462	−0.01	−0.02 to 0.01	0.144
Body mass index	0.28	−0.03 to 0.59	0.075	0.01	−0.01 to 0.02	0.354	0.02	−0.01 to 0.04	0.151
Systolic blood pressure, ±10 mmHg	0.08	−0.18 to 0.33	0.542	−**0.04**	−**0.07** to **0.01**	**0.047**	−0.03	−0.08 to 0.02	0.278
Diastolic blood pressure, ±10 mmHg	−0.04	−0.30 to 0.22	0.772	−0.01	−0.05 to 0.03	0.476	−0.02	−0.10 to 0.06	0.580
Heart rate, ±10 bpm	**0.20**	**0.09–0.32**	**0.001**	−0.01	−0.02 to 0.02	0.784	**0.03**	**0.01–0.06**	**0.022**

The *β* values and their 95% CI were obtained using a multivariate linear regression model to estimate ONSD (mm) and the RI of the ophthalmic and central retinal arteries

Highlighted values represent significant *p* values <0.05

*ONSD* optic nerve-sheath diameter, *RI* resistive index, *kg* kilograms, *mmHg* millimetres of mercury, *bpm* beats per minute

In the analysis of Doppler imaging parameters, increasing age and systolic blood pressure were associated with higher EDV (*β* = 1.06/10-years, *p* = 0.016; *β* = 0.19/10 mmHg, *p* = 0.011) and consequently lower RI of the ophthalmic artery (*β* = −0.02/10-years, *p* = 0.020; *β* = −0.04/10 mmHg, *p* = 0.016). Similarly to the ONSD, increasing heart rate was associated with higher RI of the central retinal artery (*β* = 0.03/10 bpm, *p* = 0.010). These changes withstood correction for other co-variates in the multivariate analysis (Table 4). No individual effect of demographic or clinical variables was observed on the systolic velocities of the ophthalmic vessels. Similarly, there was no significant association between the ONSD and Doppler waveform parameters (*p* > 0.05 in all comparisons).

### Effect of positioning on ONSD

**Fig. 3:**
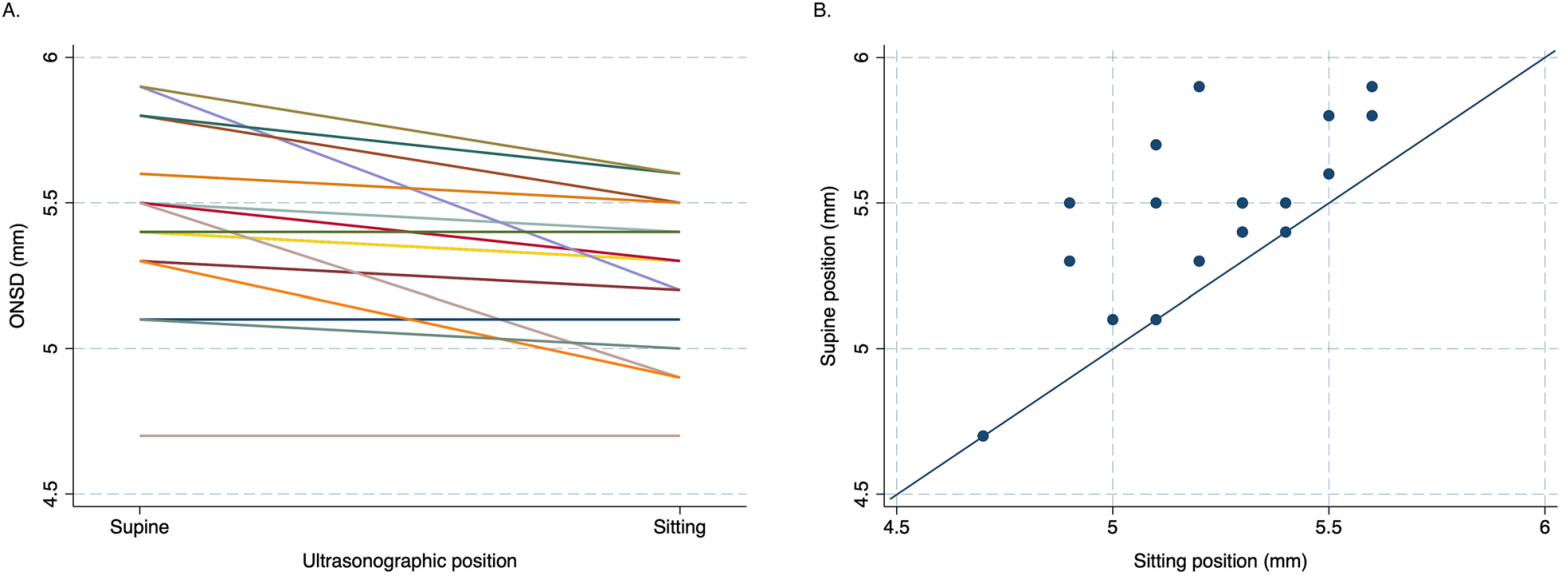
Paired supine/sitting ONSD measurements. **A** Paired observations of ONSD in supine and sitting positions showing a reduction of the ONSD in 85% of all measurements (*n* = 17) and no change in 15% of measurements (*n* = 3). **B** Bivariate plot of paired measurements showing higher ONSD in supine position compared to sitting position in most measurements (shown as points above the line of equality). The mean ONSD difference was 0.2 mm (95% Cl 0.1–0.3 mm, *p* < 0.001). ONSD, optic nerve sheath diameter; mm, millimetres

An exploratory analysis of 20 ultrasound examinations assessed the effect of raising from supine to sitting position on the ONSD in a subset of participants, with ultrasonographic measurements being taken in immediate succession on both positions. A reduction of ONSD was observed in most paired measurements (*n* = 17, 85%), and remained unchanged in the remainder 3 observations (Fig. 3). In the subgroup analysis, the mean ONSD in supine position was significantly higher (5.5 mm, 95% CI 5.3–5.6 mm) than in sitting position (5.2 mm, 95% CI 5.1–5.3 mm). Overall, assessment in sitting position resulted in a mean ONSD reduction of 0.2 mm (95% CI 0.1–0.3 mm, *p* < 0.001) compared to the supine position.

## Discussion

### Imaging features of the ONSD

Our study describes the normal imaging features of the ultrasonographic ONSD and Doppler imaging of the ophthalmic, central retinal and short posterior ciliary arteries. The mean ONSD was 5.9 mm (95% CI 5.8–6.0 mm) and both observers found similar mean ONSD measurements (rater 1: 5.93 mm; rater 2: 5.85 mm). ONSD was significantly larger in males compared to females and it increased with heart rate. Additionally, there was a significant reduction of the mean ONSD from supine to sitting position (0.2 mm, 95% CI 0.1–0.3 mm). The ICC estimates revealed a ‘good to excellent’ inter-observer reliability of the ONSD.

Previous ultrasonographic studies have reported mean ONSD in individuals with normal ICP to range between 4.0 and 6.6 mm [10, 27, 28]. Studies using magnetic resonance imaging (MRI) for greater anatomical detail have suggested a mean ONSD in healthy individuals of 5.7 mm [12, 29], which is in line with our findings. Additionally, ophthalmologic studies on intraocular pressure and normal tension glaucoma have also performed ONSD measurements and described even higher mean values in their control groups ranging from 6.1 to 6.3 mm [30, 31]. A previous anatomical study of the optic nerve on cadaver dissection performed by our research group found a mean ONSD of 5.0 mm, which we interpreted as a probable underestimation of the ONSD in vivo due to the loss of the cerebrospinal fluid surrounding the optic nerve during the dissection [32]. Paradoxically, several diagnostic studies of increased ICP set a predefined ONSD cut-off of 5.0 mm to detect intracranial hypertension [33–41]. These differences have limited the applicability of ONSD and stress the importance of defining a normal ultrasonographic ONSD. Our results in a healthy population suggest a normal mean ONSD from 5.8 to 6.0 mm, which would indicate that higher cut-off values for diagnosing increased ICP should be considered, as previously suggested [6].

### Determinants and sources of heterogeneity in ONSD assessment

The discrepancies in literature may be related to methodological differences in the assessment of ONSD, namely in the interpretations of the optic nerve sheath limits and the measurement of the dura-mater internal or external limits, which have been associated with significant variations in ONSD [13, 42]. A previous review of the role of optic nerve sheath ultrasonography also found that patient positioning and head elevation varies across studies, and, in individuals with hydrocephalus, it was suggested that upright positioning could reduce the ONSD measurement [15], however this was not observed in healthy individuals [43]. We found a previously unreported association of ONSD and subject positioning (sitting versus supine at 0°) in a healthy population, emphasizing the relevance of head elevation during ultrasonography and the importance of considering patient positioning when interpreting ONSD measurements. These findings provide a foundation for future research, particularly on the effects of varying degrees of head elevation—particularly the 30°–45° range commonly used in critical care settings—on the temporal correlation between changes in head position and adaptive ONSD responses, and on the association with variations in ICP.

The methodological variations between studies’ cohorts are likely more significant contributors to the discrepancies in the literature rather than the ultrasonographic accuracy itself. This is supported by the high inter-observer reliability we found in the ONSD assessment, consistent with previous studies using a standardized measurement technique. The documented inframillimetric agreement rates underscore the accuracy of the technique when applied to the same population [14, 44]. However, cautious interpretation should be taken when a standard technique is not employed, as inter-observer agreement drops significantly without it [45]. Therefore, variations in technique and positioning may explain some of the differences in ONSD cut-offs.

In addition to methodological variability, demographic and clinical differences may play a significant role in the definition of a normal ONSD range [43, 46]. Male sex was previously proposed as a determinant of larger ONSD, while equivocal reports exist on the effect of age [46–48]. This is in line with our findings, with significant differences between males and females. Additionally, while we found that ONSD increased with age on univariate analysis, this effect was lost in the multivariate analysis when heart rate was added as covariate. To our knowledge, this is the first report of an association between heart rate and ONSD, which could suggest an association of cardiac cycle and cerebrospinal fluid (CSF) flow into the optic nerve-sheath. Additional investigation should be conducted with synchronized heart rate monitoring and ONSD evaluation. This association could be related to the previously described direct association of heart rate and ICP [49, 50] and/or to an increased ratio of systolic to diastolic duration induced by the rising heart rate [51, 52], which could lead to more intracranial CSF outflow during systole compared to inflow during diastole [53, 54]. Further investigation into the association of heart rate and the optic nerve sheath and CSF dynamics is required.

### Imaging features of the ophthalmic vessels

Our results of Doppler imaging showed higher velocities and vascular resistance in the ophthalmic artery (PSV: 35.6 cm/s; EDV: 7.4 cm/s; RI: 0.78) and lower in the central retinal artery (PSV: 12.0 cm/s; EDV: 3.5 cm/s; RI: 0.70) and the short posterior ciliary artery (PSV: 10.2 cm/s; EDV: 3.6 cm/s; RI: 0.64). In the ophthalmic artery, increasing age and systolic blood pressure were associated with higher EDV and lower RI, while, in the central retinal artery, male sex and increasing heart rate were associated with higher RI. The ICC estimates revealed a ‘good to excellent’ inter-observer reliability of the ONSD. Estimates for the PSV of all three vessels and for EDV and RI of the ophthalmic and central arteries also showed an overall ‘good’ inter-observer reliability, while the ESA had an overall ‘poor’ and ‘moderate’ reliability. The ICC estimates of Doppler parameters had wider 95% CI compared to ICC estimates of the ONSD.

Previous studies on the Doppler imaging parameters of the ophthalmic vessels have shown conflicting results, with an older study including healthy individuals reporting a lower PSV and a higher RI in the central retinal artery compared to the ophthalmic artery, which could be driven by lower end-diastolic velocities [23]. In contrast, two later studies described higher velocities and resistive indices in the ophthalmic artery compared to the other orbital lower resistance vessels: the central retinal and short posterior ciliary arteries [24, 55], which support our own findings. The ophthalmic artery is a larger muscular artery supplying the orbit, including the extraocular muscles and connective tissue of the orbit, which have higher downstream impedance and explain its higher resistance profile. Conversely, the central retinal artery and short posterior ciliary arteries supply the inner and outer layers of the retina and the optic disk. This neural tissue has high metabolic demand and requires constant blood flow, therefore explaining the lower resistance flow of these vessels [1, 24, 56]. Studies on glaucoma and intracranial hypertension including healthy individuals revealed ranges of PSV, EDV and RI of the ophthalmic artery (PSV: 32.27–40.1 cm/s; RI: 0.73–0.82) consistent with our results [19, 22]. However, there are wide discrepancies in the reported normal PSV, EDV and RI of the central retinal artery (PSV: 8.8–17.3 cm/s; RI: 0.63–0.76) and short posterior ciliary artery (PSV: 8.6–14.2 cm/s; RI: 0.53–0.68) [24], but our findings fall within the reported ranges [21, 22]. Doppler imaging of the ophthalmic vessels may be useful to diagnose increased ICP, as the compression of intracranial vessels and higher venous pressure in these scenarios could be transmitted back to the ophthalmic artery, reducing the diastolic flow and increasing the resistance of the ophthalmic artery [57]. However, previous studies have reported both increased and decreased PSV and RI of the ophthalmic vessels in patients with intracranial hypertension and further research is necessary [20, 21]. Our suggestion of a normal range for the mean Doppler parameters of the ophthalmic vessels in healthy individuals may be an important contribution to its clinical application in the detection of increased ICP. Further studies are required to assess the vascular response of these vessels in situations of increased ICP.

### Determinants in ophthalmic vessels assessment

Demographic and clinical determinants also impacted the vascular resistance of the ophthalmic and central retinal arteries, with male sex and increasing heart rate being associated with higher RI in the central retinal artery, similarly to the ONSD findings. In the ophthalmic artery, however, EDV and RI respectively showed a direct and inverse correlation with increasing age and systolic blood pressure. Previous reports have more often found reduced velocities and increased resistance in the ophthalmic artery [58], however studies have shown very contradictory results on the effect of age, sex, heart rate and blood pressure on the Doppler parameters of the ophthalmic vessels [55, 59], so these findings should be interpreted cautiously.

Studies on the reliability of Doppler imaging of the ophthalmic vessels have previously described an overall ‘poor to moderate’ inter-observer agreement [60, 61], with one study in glaucoma patients reporting a good inter-rater reliability for the ophthalmic artery and moderate for the central retinal artery with a standardized technique. Information about normal ESA in healthy populations is missing and no previous reports exist of the mean ESA in the central retinal and short posterior ciliary arteries of a healthy population. However, while the ICC central estimates were ‘good’ for the PSV all ophthalmic vessels and for the RI of the central retinal and ophthalmic arteries, there was a ‘poor to moderate’ ICC for the ESA of all three vessels. The lower ICC estimates found in the ESA were associated with a greater variability in the data, as shown by the wider CI estimates. This could be related to discrepancies in the point of baseline and slope determination. These findings suggest that this waveform parameter should be interpreted cautiously.

### Limitations

Our study has several limitations. Firstly, ultrasonography is influenced by technical factors which as previously mentioned may impact interpretation. As such, our findings are limited by the ultrasound device and probe used and maybe differ slightly in different technical settings. Additionally, we included a sample of healthy young participants, evenly matched on sex and without vascular risk factors, which may impact the generalisability of our results as reference imaging features for older individuals. Finally, we included healthy individuals who volunteered to participate in the study which resulted in a small sample size. The small sample size may have affected the validity of our results and widened the 95% CI of our estimates, which may have reduced our power to detect differences. Despite these limitations, we were able to report consistent and reproducible imaging features in a healthy population, as well as detect a clear correlation of patient positioning with the ONSD, which supports our findings on the normal ONSD and Doppler imaging of the ophthalmic vessels, and our claim on the importance of a standardized protocol to obtain reproducible results. This study provides preliminary results on the impact of demographic and methodological factors to guide future research on this topic.

## Conclusions

Our findings support a larger mean ONSD as a reference standard and may indicate that higher cutoffs to detect increased ICP should be used. Demographic and clinical variables and positioning during ultrasonography impact the measurements of the ONSD and Doppler parameters of the ophthalmic vessels. Overall, ultrasonography is a widespread method to assess the ONSD and Doppler waveform parameters of the ophthalmic and central retinal arteries, which can inform intracranial pressure. However careful attention should be given to the measurement technique, specifically the positioning during the examination, which can markedly affect accuracy and reliability of optic nerve ultrasonography.

## Data Availability

The datasets used and/or analysed during the current study are available from the corresponding author on reasonable request.
